# Process Investigation on Robust Electrospinning of Non-Aligned and Aligned Polyvinylidene Fluoride Nanofiber Mats for Flexible Piezoelectric Sensors

**DOI:** 10.3390/polym16060816

**Published:** 2024-03-14

**Authors:** Xiaohua Liu, Minghai Zhang, Baolin Jiang, Qihao Zhang, Hao Chen, Yan Shen, Ziyan Wang, Xiaohong Yin

**Affiliations:** 1 College of Urban Transportation and Logistics, Shenzhen Technology University, Shenzhen 518118, China; liuxiaohua@sztu.edu.cn (X.L.); 2210414003@stumail.sztu.edu.cn (M.Z.); 202200401031@stumail.sztu.edu.cn (B.J.); 2310414013@stumail.sztu.edu.cn (Q.Z.); 2310414011@stumail.sztu.edu.cn (H.C.); 202100401060@stumail.sztu.edu.cn (Y.S.); 202100401051@stumail.sztu.edu.cn (Z.W.); 2 Guangdong Rail Transit Intelligent Operation and Maintenance Technology Development Center, Shenzhen 518118, China

**Keywords:** PVDF nanofiber mats, electrospinning, process parameters, flexible piezoelectric sensor

## Abstract

Polyvinylidene fluoride (PVDF) nanofiber mats have played a significant role in wearable electronic devices that have been in great demand in recent decades. Although manifold PVDFbased freely stacked or well-aligned nanofiber mats created via the electrospinning process have been demonstrated to achieve multisensory capabilities with high sensitivity and long detection range, rarely have any of them proved their ability with a stable process and accurate processing parameters. In this work, we successfully developed freely stacked and well-aligned PVDF nanofiber mats with diameters ranging from micrometers to nanometers, providing stable performance for wearable electronic devices. Through in-depth investigations into material preparation, electrospinning, and fiber collection processes, we revealed the relationship between the nanofiber morphology, β-phase fraction, and piezoelectric output with various process parameters. Characterized by analytical methods, we have established a mature, reliable nanofiber mat fabrication system capable of mass-producing PVDF nanofibers with the required diameter and consistent properties. At 18 kV voltage and 60% RH humidity, the uniformity of the fiber diameter and β-phase content was maintained in a favorable range. When the drum speed increased to 2000 r/s, the fiber orientation and β-phase content increased. We assembled aligned PVDF nanofiber mats with conductive fabric in a flexible piezoelectric sensor that successfully monitored different body movements and produced an output voltage of 0.1 V. This study provides the necessary process parameters for the large-scale production of high-quality PVDF nanofiber mats and provides clear guidance for beginners in the field of nanofiber mat manufacturing.

## 1. Introduction

Flexible and wearable piezoelectric sensors, distinguished by their remarkable ability to convert subtle deformation into electricity, have emerged as highly promising technological solutions with a myriad of potential applications in healthcare monitoring [1,2], human–machine interfaces [3,4], biomechanics and smart sports science [5], and security and surveillance [6]. Nanofiber mats, composed of interconnected nanoscale fibers generally ranging from hundreds of nanometers to sub-micrometers in diameter, serve as advantageous components in the construction of piezoelectric sensors with their extraordinary responsiveness to mechanical stimuli, such as pressure, strain, or vibrations, and perfect flexibility and inherent mechanical strength properties [7,8]. These mats can be fabricated by using various techniques, including electrospinning [9], solution blow spinning [10], and so on. Specifically, electrospinning stands out as a versatile and widely used nanofabrication technology that uses electrostatic forces to stretch the polymer solution or melt it into extremely fine fibers and then deposits these fibers onto a collector to form nanofiber mats [9]. Among the many materials adopted for nanofiber mats, PVDF, a semi-crystalline polymer, has excellent chemical stability, piezoelectric properties, and a high dielectric constant, making it an ideal candidate material for electrospinning [11,12]. In the pursuit of improving the performance and functionality of PVDF nanofiber mats through the electrospinning process, researchers have discovered that the morphology and arrangement of PVDF nanofibers can be precisely controlled into two styles: non-aligned and aligned nanofiber structures.

Non-aligned PVDF fibers exhibit a randomly arranged nanofiber structure, showcasing unique isotropic properties. The nanofibers in this material are uniformly and randomly distributed within the mats, imparting similar physical properties in all directions. This non-aligned nature provides PVDF nanofiber mats with broad applicability, particularly in scenarios where a specific directional alignment is unnecessary, as in biomedical applications like cell diaphragms [13]. In these applications, the isotropic nature of the mats ensures the uniform blocking or transmission of substances in all directions. Additionally, it finds applications in fields such as piezoelectric sensors [14,15,16,17], helping to avoid performance variations in specific directions

The aligned PVDF nanofiber mats exhibit a well-defined arrangement of nanofibers along a single direction, typically achieved by adjusting the collector’s collection method or the size and layout of the external electric field. Commonly used electrospinning collectors include static collectors (metal plate collectors [18,19], double-plate collectors [20,21], circle electrode collectors [22]), and rotating collectors (drum collectors [23,24]). In comparison to static collectors, rotating collectors provide an additional stretching force during the electrospinning process, stretching the obtained PVDF nanofibers. When the rotating collector reaches a certain rotational speed, it assists in aligning the fibers, inducing the polarized orientation of PVDF. In general, compared to static collectors, rotating collectors are more conducive to the preparation of oriented PVDF fibers. Aligned PVDF fiber mats with specific orientations demonstrate excellent electromechanical properties [25]. Even at very low pressure values (0.1 Pa), they achieve ultra-high measurement sensitivity [26]. The electrical output of the PENG based on aligned fiber webs surpasses that of the PENG based on randomly aligned fiber webs [27].

Although non-aligned and aligned PVDF nanofiber mats have been extensively studied, the relationship between the spinning parameters, morphology, and piezoelectric performance of both fiber structures has not been systematically studied. This study aims to delve into the basic principles governing the formation of PVDF nanofibers during the electrospinning process and investigate how various process parameters (voltage, humidity, and collector (drum) speed configuration) affect the morphology, arrangement, and piezoelectric properties of the resulting nanofibers. Using scanning electron microscopy to observe the fiber morphology under different spinning parameters and analyze the impact of the process parameters on the fiber morphology. The β-crystalline phase content of different PVDF fibers was analyzed using X-ray diffraction and Fourier-transform infrared spectroscopy, and the influence of the process parameters on the crystalline phase structure was investigated. The piezoelectric properties of the mats under different spinning parameters were observed using a vibrator and oscilloscope, and the influence of the process parameters on the piezoelectric performance was analyzed. By elucidating these key factors, we aim to improve the understanding of the electrospinning process and provide valuable insights into the design and optimization of PVDF nanofibers with tunable nanofiber sizes, nanofiber arrangements, and remarkable piezoelectric properties.

## 2. Fabrication of Pvdf Nanofiber Mats and Pressure Sensor

### 2.1. Material and Solvent Preparation

**Figure 1:**
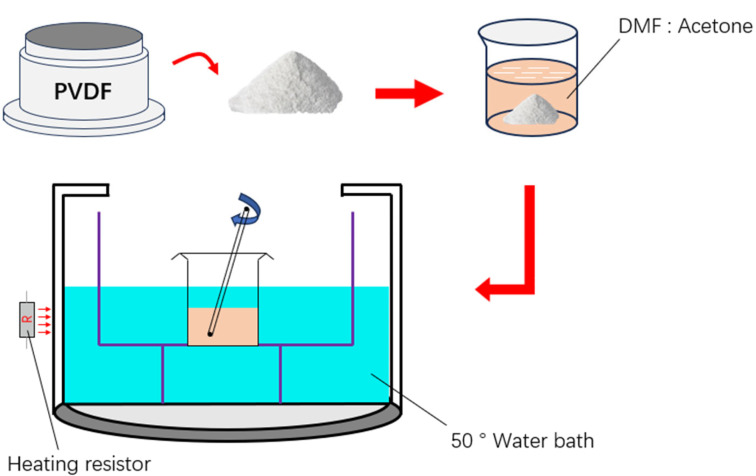
Schematic of electrospinning solvent preparation process.

To achieve both PVDF aligned and non-aligned nanofiber mats, the PVDF solvent needs to be prepared before electrospinning. The preparation process is shown in Figure 1. N,N-dimethylformamide (DMF) was purchased from Shanghai Boer Chemical Reagent Co., Ltd., (Shanghai, China). PVDF powder (*M_w_* ≈ 300,000) was purchased from Dongguan Zhan Yang polymer materials Co., LTD., (Dongguan, China). Initially, DMF and acetone are mixed in a mass ratio of 6:4 to create a mixed solvent, chosen as the medium for dissolving the polymer. Subsequently, PVDF powder with a mass fraction of 20 wt% has been gradually added to the mixed solvent to gain complete dissolution of PVDF powder. To guarantee thorough mixing, the mixture is then transferred to a water bath set at 50 °C and continuously stirred for 2 h. During this process, the temperature and stirring time are two significant factors that determine the full interaction between PVDF powder and the mixed solvent, ultimately resulting in a homogeneous PVDF electrospinning solution. Proper parameters during this preparation process are crucial to ensure the successful production of high-quality nanofibers.

### 2.2. Electrospinning Process under Different Conditions

**Figure 2:**
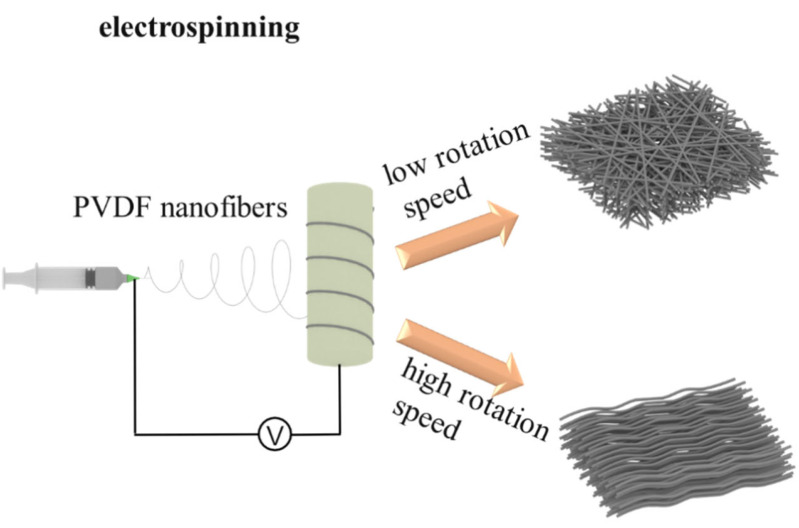
Electrospinning schematic diagram.

To investigate the complex interactions among the processing parameters, experiment setup layout and arrangement, and the formation morphology, dimensions, and performance of fibers, several groups of electrospinning experiments were carried out under different conditions. The electrospinning process is shown in Figure 2. Compared with properties and parameters including the concentration, solvent type, solution viscosity, spinning distance, deposition rate, and collector shape of the PVDF solution, processing parameters such as voltage, environmental humidity, and drum speed of the collector seem to have a greater impact on the properties of the fibers [9]. Therefore, experiments were conducted concentrating on these three factors with other fixed properties and parameters. Nine sets of control experiments were conducted, as shown in Table 1.

### 2.3. Electronic Skin Pressure Sensor Assembly

**Figure 3:**
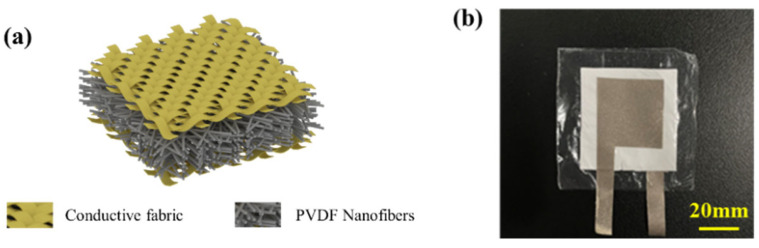
Electronic skin single-unit structure: (**a**) schematic diagram; (**b**) physical image.

The proposed fiber-based electronic skin in this work comprises one encapsulation layer, one piezoelectric layer, and upper and lower conductive fabrics, as shown in Figure 3a–b. In the preparation process, the piezoelectric mats were first precisely cut to dimensions of 3 cm × 3 cm to ensure coordinated compatibility with subsequent components. Subsequently, conductive fabrics were adhered to both sides of the electrospun piezoelectric mats, serving as electrodes at both ends. The area of these conductive fabrics was restricted to be smaller than that of the piezoelectric mats, ensuring that it does not compromise the sensitivity of the electronic skin. Finally, for comprehensive external protection of the electronic components, two larger pieces of PU mats were chosen and attached to the top and bottom of the electronic skin, forming a robust encapsulation structure. This meticulous assembly process aims to ensure the reliability and stability of the electronic skin components in complex environments while providing ample protection for outstanding performance in practical applications.

### 2.4. Characterization and Measurement

The piezoelectric properties of PVDF nanofiber mats mainly depend on their arrangements of molecular chains consisting of repeating units (-CH_2_-CF_2_-) containing carbon, fluorine, and hydrogen atoms. In the composition of the PVDF molecular chain, fluorine atoms have high electronegativity, while hydrogen and carbon atoms have low electronegativity. Therefore, the PVDF molecular chain is polar, with the fluorine end (CF_2_) enriched in electronegativity, and the hydrogen end enriched in electronegativity. Different arrangements of molecular chains create different crystal phases. There are currently five known crystal phases, namely α, β, γ, δ, and ε [28]. The most common crystal phases are α phase and β phase. The existence and relative proportion of the crystal phase structure also have an important impact on the performance and application of PVDF. The α phase is a typical crystal phase structure of PVDF, which has high symmetry and weak piezoelectric properties. The β phase is formed under stretching or electric field and has lower symmetry and good piezoelectric properties [29,30]. The formula for calculating β-phase content is as follows [31]:

(1) $$F_{\beta }=\frac{A_{\beta }}{\frac{K_{\beta }}{K_{\alpha }}A_{\alpha }+A_{\beta }}\times 100\%$$

A_α_ and A_β_ are the absorbance at 766 cm^−1^ and 840 cm^−1^, respectively; K_α_ (6.1 × 104 cm^2^·mol^−1^) and K_β_ (7.7 × 104 cm^2^·mol^−1^) are the absorption coefficients at 766 cm^−1^ and 840 cm^−1^ [31].

Therefore, Fourier-transform infrared spectroscopy (FT-IR) spectral analysis is performed using spectrometers (Nicolet iS50, Thermo Fisher scientific, Waltham, MA, USA) to detect the crystal phase structure of the fiber mats. FTIR measurements were performed on different piezoelectric mats. The X-ray diffractometer (SmartLab XRD, Rigaku Corporation, Tokyo, Japan) was used to analyze the crystal phase structure of the fiber mats. The sample is scanned within the range of 10–80°. Apart from this, electrical testing of electronic skin units was also conducted by using exciters and an oscilloscope to present the piezoelectric properties of fabricated nanofiber mats.

In addition, the morphology and mechanical performance of the nanofiber mats were characterized as well. A field emission scanning electron microscope (GeminiSEM 300, Carl Zeiss Microscopy Ltd., Cambridge, UK) was used to observe the appearance and diameter of different PVDF fibers. Before observation, conductive adhesive was used to affix the sample to the sample stage, and the corresponding gold spraying treatment was performed. The voltage observed was 15 KV. Mechanical testing of the mats was conducted using the Nanoscience Instruments FlexTest TM-L apparatus (Hunan NanoUp Electronics Technology Co., Ltd., Changsha, China).

## 3. Results and Discussion

### 3.1. Effect of Processing Parameters on Morphology and Performance of Nanofibers

#### 3.1.1. Voltage

As the primary driving force in electrospinning, voltage has a significant impact on the fiber formation process. Moderate voltage levels contribute to a more uniform distribution of fiber diameters. However, excessively high voltage may lead to fiber breakage, limiting continuous production. Therefore, precise voltage is a crucial factor in ensuring fiber quality and production efficiency in the electrospinning process.

**Figure 4:**
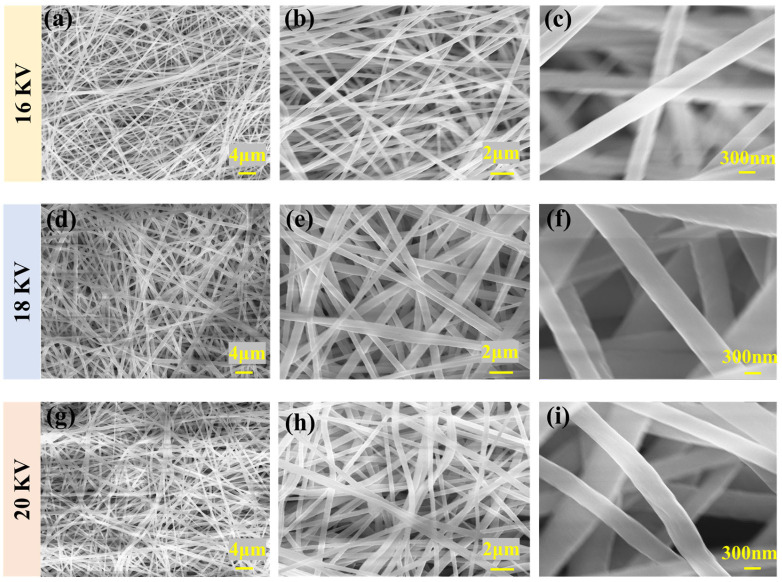
SEM images of PVDF nanofibers under different voltages: (**a**) 16 KV (2000 times magnification), (**b**) 16 KV (5000 times magnification), (**c**) 16 KV (25,000 times magnification), (**d**) 18 KV (2000 times magnification), (**e**) 18 KV (5000 times magnification), (**f**) 18 KV (25,000 times magnification), (**g**) 20 KV (2000 times magnification), (**h**) 20 KV (5000 times magnification), and (**i**) 20 KV (25,000 times magnification).

**Figure 5:**
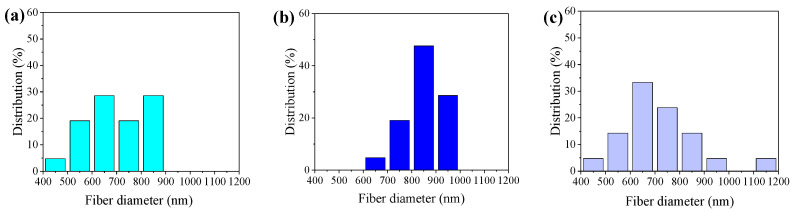
Diameter distribution of the PVDF (**a**) 16 KV, (**b**) 18 KV, and (**c**) 20 KV.

As shown in Figure 4, the fiber diameter exhibits a noticeable decreasing trend with a gradual increase in voltage under environmental conditions with relative humidity of 60%, and drum rotation speed of 625 r/min. We observed a close correlation between the uniformity of the fiber diameter and changes in voltage, as shown in Figure 4a,d,g. Specifically, as shown in Figure 4a–c, the fiber arrangement is looser. As the voltage increases, the distribution of fiber diameters becomes more uniform, as shown in Figure 4d–f, demonstrating more consistent characteristics. With the further increase in voltage, as shown in Figure 4g–i, the fiber appears to bend differently, and the fiber consistency decreases. Specifically, as shown in Figure 5a–c and Table 2, when the voltage is 18 KV, the fiber distribution is relatively concentrated, and when the voltage is reduced or increased, the random fiber distribution increases.

The β-phase content of PVDF was preliminarily and qualitatively analyzed. Figure 5a shows the X-ray diffraction (XRD) pattern of PVDF. It can be seen that at 20.1°, there is a strong diffraction peak, indicating that PVDF is polarized. For a more in-depth understanding of the β phase in PVDF, qualitative and quantitative analysis can be conducted using FTIR, as shown in Figure 5b. In the FTIR spectrum, the characteristic absorption peak for the α phase is around 763 cm^−1^, while for the β phase, it is around 840 cm^−1^.

**Figure 6:**
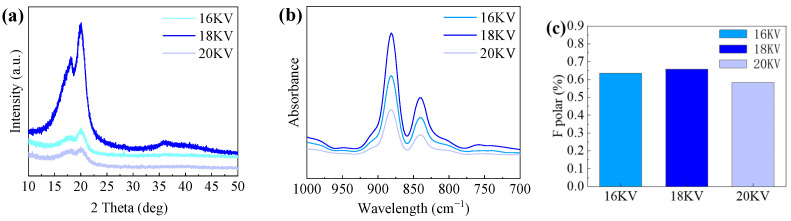
Structure analysis of piezoelectric nanofibers in voltage control group: (**a**) XRD analysis of PVDF piezoelectric fiber, (**b**) FT-IR spectra of PVDF, and (**c**) β-phase content diagram.

At 840 cm^−1^, PVDF at different voltages presents a strong diffraction peak, which is consistent with the results in Figure 6a. Figure 6c clearly shows that the voltage level has a significant impact on the β-phase content of PVDF. When the voltage is set at 16 KV, 18 KV, and 20 KV, the β-phase content of PVDF can be calculated as 63%, 65%, and 58%, by Equation (1), respectively. We can see that the β-phase content of PVDF goes up and then falls with an increase in voltage. The reason is that the molecular arrangement under different voltage conditions may significantly influence the formation of the β-phase in PVDF. With an increase in voltage, molecules are more prone to align in a more ordered manner, which is beneficial for improving the crystal structure of PVDF. Nevertheless, at an extremely high voltage, due to excessive thermal energy, the material may undergo a melting process or experience structural damage, potentially resulting in a decrease in crystallinity.

Therefore, a suitable voltage needs to be selected to achieve a balance between the enhancement of crystallinity and production stability.

#### 3.1.2. Humidity

Humidity is another important influencing factor. Increased humidity contributes to enhancing the tensile properties of fibers within a certain range. However, both excessively high and low humidity levels may result in uneven fibers or clustering. Variations in humidity also affect the viscosity of the solution, thereby influencing the formation speed and diameter distribution of fibers.

Humidity plays a significant regulatory role in fiber formation throughout the entire spinning process, and this influence is often closely tied to voltage levels. Generally, low-humidity environments are more prone to generating static electricity. In environments with low humidity, the air moisture content is relatively low, thereby increasing the likelihood of static electricity generation. As the spinning equipment operates, fibers or droplets experience friction with the surrounding air during the spraying and stretching processes, leading to the generation of static electricity.

To ensure that the spinning solution can adequately form nanofibers, it is often necessary to elevate the voltage level. However, the generation of static electricity can cause oscillation of the Taylor cone at high voltage, or even prevent the formation of a Taylor cone, thereby reducing the overall stability of the spinning process.

**Figure 7:**
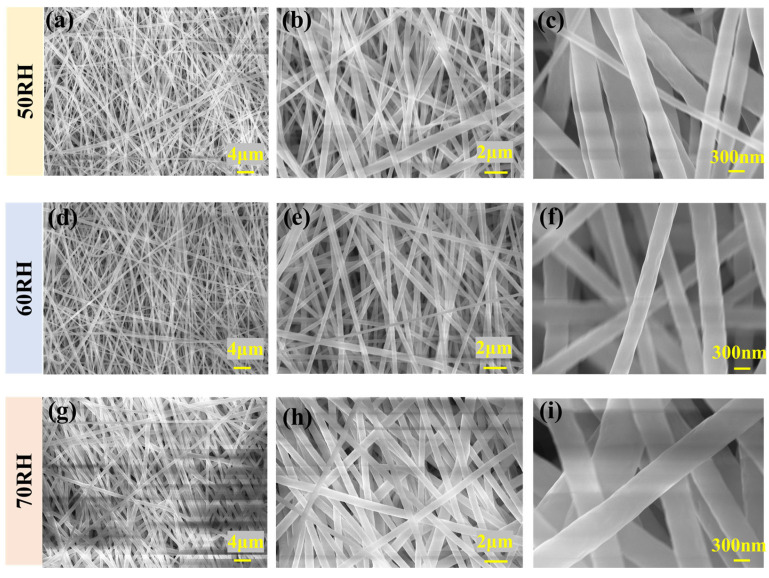
SEM images of PVDF nanofibers under different humidity: (**a**) 50 RH (2000 times magnification), (**b**) 50 RH (5000 times magnification), (**c**) 50 RH (25,000 times magnification), (**d**) 60 RH (2000 times magnification), (**e**) 60 RH (5000 times magnification), (**f**) 60 RH (25,000 times magnification), (**g**) 70 RH (2000 times magnification), (**h**) 70 RH (5000 times magnification), and (**i**) 70 RH (25,000 times magnification).

**Figure 8:**
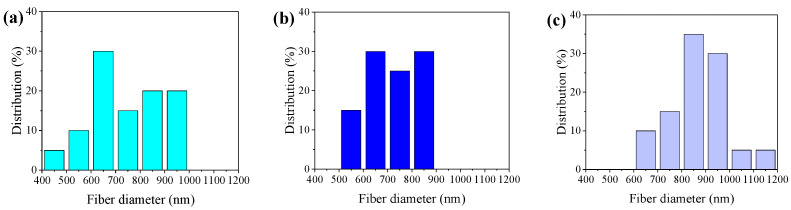
Diameter distribution of the PVDF (**a**) 50 RH, (**b**) 60 RH, and (**c**) 70 RH.

In the humidity control experiment, variations in humidity significantly impact the electrospinning process. As shown in Figure 7a–f, with increasing humidity levels, there is a noticeable reduction in fiber diameter accompanied by an enhancement in diameter uniformity. With the further increase in humidity, Figure 7g–i show that the diameter of the fibers increases significantly. In high-humidity conditions, the solvent evaporation rate of the fiber solution decreases, allowing the fibers more time for stretching and resulting in an increase in the fiber diameter. Figure 8a–c also support the above conclusions. Combined with Table 3, it is further demonstrated that humidity also has a certain influence on the uniformity of fibers.

**Figure 9:**
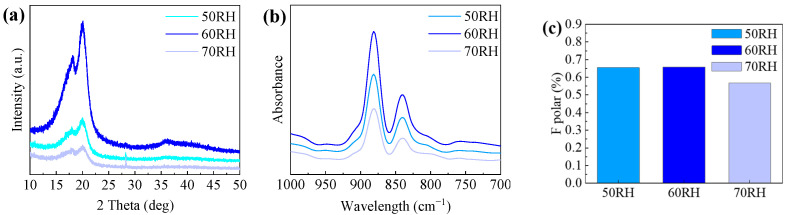
Structure analysis of piezoelectric nanofibers in humidity control group: (**a**) XRD analysis of PVDF piezoelectric fiber, (**b**) FT-IR spectra of PVDF, and (**c**) β-phase content diagram.

As shown in Figure 9a–c, under specific humidity conditions, such as 50 RH, 60 RH, and 70 RH, distinct variations in the PVDF β-phase content are observed, registering values of 64%, 65%, and 57%, respectively. In lower humidity conditions, the detrimental effects of static electricity contribute to a decrease in the crystallinity of PVDF. Conversely, excessively high humidity levels may cause the spinning material to absorb moisture, inducing a softening effect. This softening tendency may make the fibers more susceptible to deformation and elongation, thereby compromising the stability of fiber morphology and resulting in a reduction in crystallinity.

Therefore, by adjusting parameters such as humidity and voltage, it is possible to more precisely control the formation of nanofibers, providing crucial experimental results for optimizing the spinning process and enhancing fiber quality.

#### 3.1.3. Drum Speed

Drum rotation speed directly affects the stretching and solidification processes of fibers. Higher drum rotation speeds assist in stretching fibers, leading to the formation of finer fiber diameters. Nevertheless, excessively high speeds may cause fiber breakage or uneven stretching.

After conducting two sets of control experiments on voltage and humidity, we concluded that the optimal settings for spinning were 18 KV and 60 RH, respectively. Under these conditions, we varied the rotation speed of the drum to investigate the aligned behavior of the fibers.

**Figure 10:**
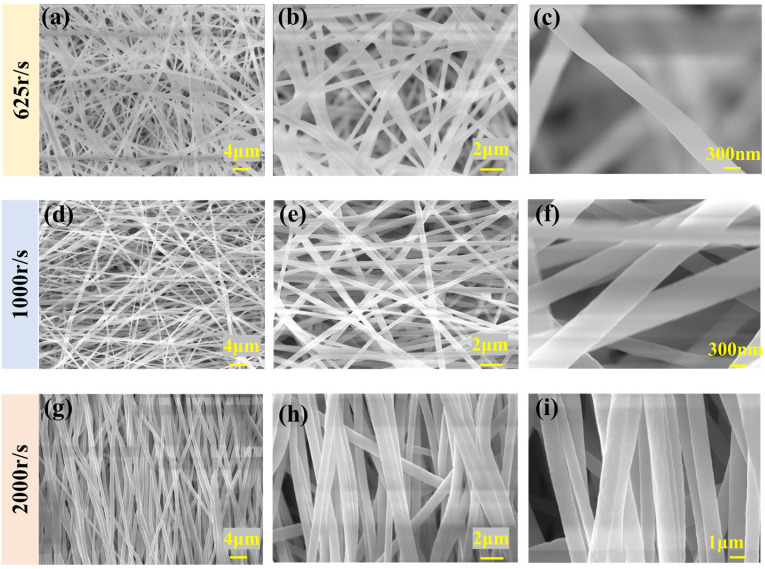
SEM images of PVDF nanofibers under different drum speed: (**a**) 625 r/s (2000 times magnification), (**b**) 625 r/s (5000 times magnification), (**c**) 625 r/s (25,000 times magnification), (**d**) 1000 r/s (2000 times magnification), (**e**) 1000 r/s (5000 times magnification), (**f**) 1000 r/s (25,000 times magnification), (**g**) 2000 r/s (2000 times magnification), (**h**) 2000 r/s (5000 times magnification), and (**i**) 2000 r/s (12,000 times magnification).

During the electrospinning process, the solution forms a Taylor cone under the guidance of an electric field, subsequently evolving into nanofibers. These nanofibers converge towards the drum at a high velocity. When the rotation speed of the drum matches the ejection speed of the nanofibers, there is no relative speed between the nanofibers and the drum, allowing the fibers to adhere to the drum in a fixed direction. This results in the aligned arrangement of fibers. As shown in Figure 10g–i, the evident fiber orientation behavior is typically observed when the drum speed is at least 2000 r/s. In contrast, as shown in Figure 10a–f, when the drum speed is 625 r/s or even 1000 r/s, the fibers exhibit a non-aligned pattern.

**Figure 11:**
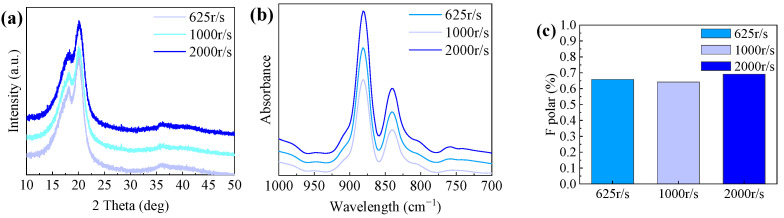
Structure analysis of piezoelectric nanofibers in drum speed control group: (**a**) XRD analysis of PVDF piezoelectric fiber, (**b**) FT-IR spectra of PVDF, and (**c**) β-phase content diagram.

Through qualitative analysis using XRD and qualitative and quantitative analysis of FTIR images, as shown in Figure 11a–c,we ultimately obtained the β-phase content of nanofibers formed at different drum speeds, measuring 65%, 64%, and 69%, respectively. This confirms that aligned nanofibers exhibit superior crystallinity, and the β-phase content can be significantly enhanced.

### 3.2. Electronic Skin Pressure Sensor Unit Performance Test

**Figure 12:**
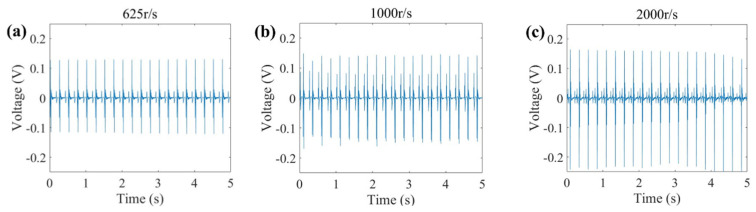
Electrical output properties of piezoelectric fibers: (**a**) PVDF (drum speed is 625 r/s), (**b**) PVDF (drum speed is 1000 r/s), and (**c**) PVDF (drum speed is 2000 r/s).

We conducted a comprehensive study on the electrical performance of electronic skin prepared using piezoelectric fiber mats at different drum rotation speeds. The applied test pressure was 30 N, with a frequency of 4 Hz. As shown in Figure 12a–c, at a low drum rotation speed, the output voltage reaches 0.12 V; with the gradual increase in drum rotation speed, the output voltage also increases, reaching 0.25 V at a high drum rotation speed. This trend aligns with the results in Section 3.3 regarding XRD and FTIR. We attribute the enhanced piezoelectric performance to the transformation of the internal phase structure of PVDF. This further confirms that higher rotation speeds contribute to fiber alignment and facilitate the phase transition from the α phase to the β phase within PVDF.

**Figure 13:**
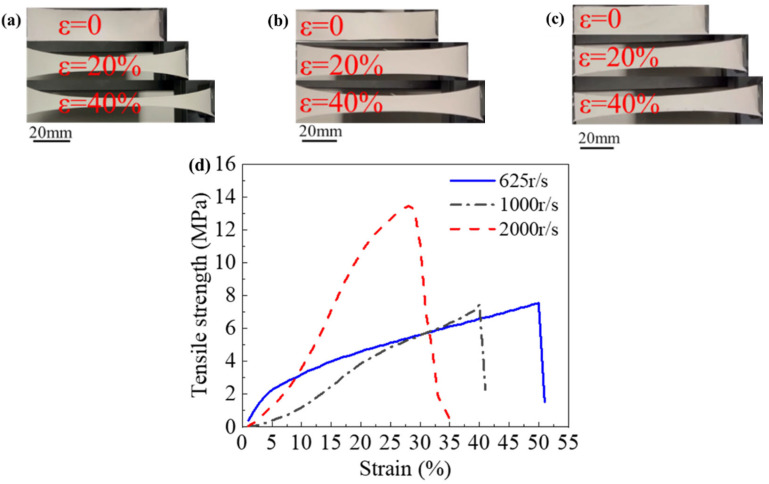
(**a**) Tensile performance demonstration of 625 r/s mats, (**b**) tensile performance demonstration of 1000 r/s mats, (**c**) tensile performance demonstration of 2000 r/s mats, and (**d**) the mechanical properties of different nanofiber mats.

We systematically investigated the mechanical properties of piezoelectric thin mats at three different rotational speeds. As shown in Figure 13a–c, at low rotational speeds, the thin mats exhibited excellent ductility. Particularly noteworthy is that, compared to the ordered arrangement of fiber structures, randomly arranged fibers demonstrated superior performance under tensile forces. This observation is not only associated with the morphology of the structure but also involves the overall response of the material to external stresses. The randomly arranged fiber structure possesses characteristics of multi-aligned stress distribution, facilitating the uniform dispersion of external stresses in multiple directions. This feature effectively mitigates stress concentration phenomena, thereby enhancing the overall tensile performance of the material. In contrast, the aligned structures have higher tensile strength, as shown in Figure 13d.

**Figure 14:**
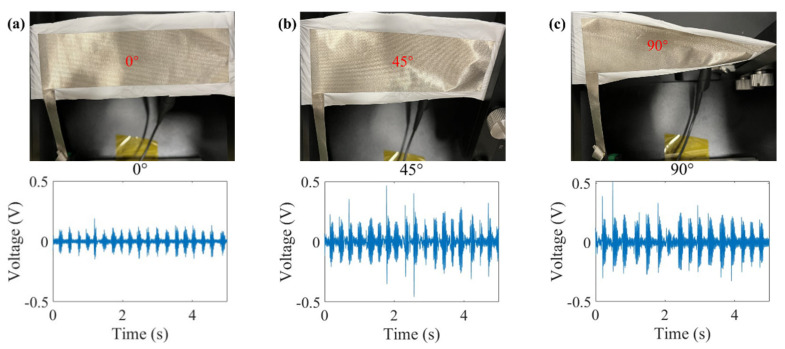
Electrical performance test of PVDF at different rotation angles: (**a**) 0°, (**b**) 45°, (**c**) 90°.

**Figure 15:**
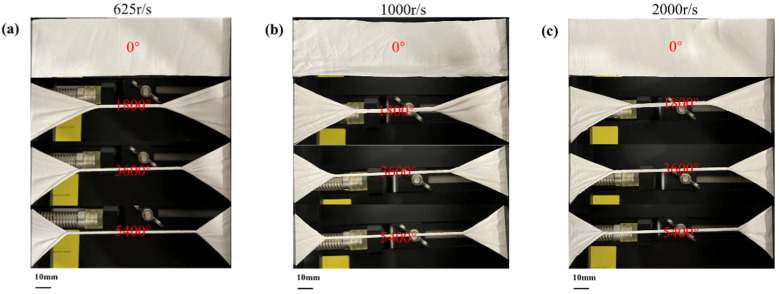
Twist diagram of PVDF with different parameters: (**a**) 625 r/s, (**b**) 1000 r/s, and (**c**) 2000 r/s.

In addition, the torsional properties of the mats were tested. Figure 14a–c, respectively, show that PVDF mats can still output electrical properties under torsion angles of 0°, 45°, and 90°, and the angle will not affect the electrical output basically. We also tested the torsional strength of the mats, as shown in Figure 15a–c. The results showed that the mats with different surface parameters had good torsional resistance and would not break when 5400° was selected.

### 3.3. Human Joint Movement Characterization by Flexible Sensors

**Figure 16:**
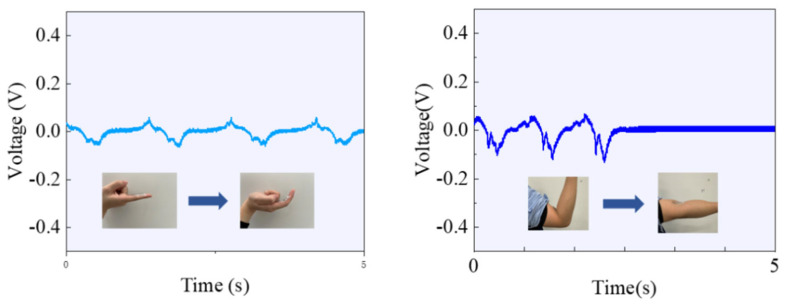
Schematic diagram of electronic skin sensing joint movement.

We further explored the practical application of flexible sensors in sensing human joint movements. Initially, by using PU tape, this electronic skin can be seamlessly adhered to joint locations such as fingers and elbows. As shown in Figure 16, it accurately expresses the amplitude and frequency of the corresponding joint movements in the form of varying signal strength. When the elbow joint is extended and not bent, the electronic skin does not output any signals; however, with an increase in the degree of elbow joint flexion, the output signals of the electronic skin also increase. Therefore, by using PU tape, the electronic skin can achieve highly sensitive monitoring of joint movements in locations like fingers and elbows without relying on an external power source. Additionally, this adaptive electronic skin not only efficiently perceives joint movements but also possesses the capability to adapt to different postures. Overall, our developed electronic skin offers outstanding performance and extensive application prospects for achieving highly flexible joint movement monitoring.

## 4. Conclusions

In summary, this work elaborately presents electrospinning processes under several groups of significant processing parameters, including voltage, humidity, and drum rotation speed, and compares these fabricated non-aligned and aligned PVDF nanofiber mats in electric and mechanical properties. The following conclusions have been drawn:

(1) Both the uniformity of fiber diameter and the content of the β phase can be maintained within a favorable range when the voltage is 18 kV and the humidity is 60% RH. Building upon this foundation, the fibers exhibited significant orientation, and the content of the β phase showed a noticeable increase when the drum rotation speed reached 2000 r/s.
(2) Higher drum rotation speed leads to aligned piezoelectric nanofibers, which exhibit higher electrical and mechanical output performance compared to non-aligned piezoelectric fibers.
(3) The wearable flexible sensor can maintain good electrical output at a distortion angle of 0–90° and has good torsional resistance.
(4) The prepared wearable flexible sensor exhibits high shape adaptability without compromising its sensing capabilities. Simultaneously, its self-powered functionality eliminates the need for rigid batteries, allowing it to conform well to curved surfaces such as joints for sensing.

## Figures and Tables

**Table 1:** Voltage, humidity, and drum speed control experiment.

	Voltage	Humidity			Drum Speed	Deposition Rate
Voltage control group		Set value	Mean	Variance	625 r/s	0.36 cm^3^/h
	16 KV	50 RH	49.98 RH	1.06		
	18 KV	50 RH	49.58 RH	1.36		
	20 KV	50 RH	50.01 RH	1.09		
Humid control group	18 KV	Set value	Mean	Variance	625 r/s	0.36 cm^3^/h
		50 RH	50.04 RH	1.14		
		60 RH	59.51 RH	1.09		
		70 RH	69.93 RH	0.80		
Rotation speed control group	18 KV	Set value	Mean	Variance		0.36 cm^3^/h
		60 RH	59.51 RH	1.09	625 r/s	
		60 RH	59.56 RH	1.11	1000 r/s	
		60 RH	59.58 RH	0.99	2000 r/s	

**Table 2:** Averages and variances at different voltages.

Voltage	Average (μm)	Variance
16 KV	0.689	0.0151
18 KV	0.847	0.007
20 KV	0.709	0.0278

**Table 3:** Averages and variances at different humidity.

Humidity	Average (μm)	Variance
50 RH	0.743	0.0230
60 RH	0.718	0.0114
70 RH	0.875	0.0142

## Data Availability

Data are contained within the article.
